# Mobile-Health Technologies for a Child Neuropsychiatry Service: Development and Usability of the Assioma Digital Platform

**DOI:** 10.3390/ijerph18052758

**Published:** 2021-03-09

**Authors:** Elisa Fucà, Floriana Costanzo, Dimitri Bonutto, Annarita Moretti, Andrea Fini, Alberto Ferraiuolo, Stefano Vicari, Alberto Eugenio Tozzi

**Affiliations:** 1 Child and Adolescent Neuropsychiatry Unit, Department of Neuroscience, Bambino Gesù Children’s Hospital, IRCCS, 00165 Rome, Italy; elisa.fuca@opbg.net (E.F.); stefano.vicari@opbg.net (S.V.); 2 Multifactorial and Complex Disease Research Area, Bambino Gesù Children’s Hospital IRCCS, Piazza S. Onofrio 4, 00165 Rome, Italy; dimitri.bonutto1@gmail.com (D.B.); albertoeugenio.tozzi@opbg.net (A.E.T.); 3 Topnetwork S.p.A, 00142 Rome, Italy; annarita.moretti@top-network.it (A.M.); finello76@gmail.com (A.F.); alberto.ferraiuolo@top-network.it (A.F.); 4 Department of Life Science and Public Health, Catholic University of the Sacred Heart, 00168 Rome, Italy

**Keywords:** mHealth, child neuropsychiatry, day hospital

## Abstract

We developed an m-Health platform to support clinical pathways in a child and adolescent neuropsychiatry unit. The Assioma platform was created for tablets, smartphones and PCs, to support data collection and clinical workflow, to promote constant communication between patients, caregivers and clinicians, and to promote active family involvement in day hospital (DH) procedures. Through the Assioma application for tablets, caregivers filled out an anamnestic questionnaire and explored contents on the DH procedures and neurodevelopmental conditions. The application for smartphones included an agenda function for the DH pathways. Through the application for desktops, clinicians could export anamnestic information in text and Excel formats, send real-time notifications, and push relative contents to families’ account. We tested the usability and satisfaction of the Assioma platform in a group of children, caregivers (N = 24) and clinicians (N = 6). Both families and clinicians gave high scores to almost all usability items. The overall satisfaction reached the highest levels at 50% satisfied for families and at 33% for clinicians. Our results indicate that the Assioma platform has the potential to optimize clinical pathways, increasing compliance and clinical efficiency, and to reduce in-person contacts supporting social distancing for clinical pathways, a crucial need during the COVID-19 pandemic.

## 1. Introduction

The expression “Mobile health” (m-Health) refers to health services supported by mobile communication devices, such as patient monitoring, alert systems, data collection, record maintenance, and prevention systems [1]. They are designed to target specific health topics, such as the prevention of infectious diseases [2], and symptom management for different medical conditions [3,4]. M-Health technologies can also support the organization of healthcare services: they have the potential to be used to optimize clinical pathways by supporting the collection of clinical histories, facilitating long-term clinical data storage and sharing across institutions [5]. Overall, m-Health technologies have the potential to improve workflow, the quality and efficiency of communication, and accessibility as well as inter-team relationships within healthcare services [6].

An increasing amount of research has been focused on the application of telemedicine and m-Health systems for mental health [7], including interventions for children [8]. M-Health technologies for mental health for pediatric population have been developed with different purposes: health promotion [9], prevention [10,11], symptoms monitoring [12,13], treatment [14,15], the monitoring of, and supporting adherence to, pharmacological and behavioral treatments [16,17]. 

In Italy, child and adolescent neuropsychiatry services manage diagnosis, rehabilitation, and intervention needs of children with neurodevelopmental and/or psychiatric disorders. These services are faced with many challenges such as the increasing number of patients, the lack of an informative and monitoring system and the high heterogeneity in the services provided across different geographic areas [18]. Thus, the need for resource optimization among child and adolescent neuropsychiatric services has strongly emerged. 

M-Health technologies may contribute to addressing these issues: they have the potential to help improving organization and efficiency in mental healthcare services. For example, mobile communication devices allow for the collection of extremely rich data and can help by automatically organizing and storing the data. Another potential contribution of m-Health technologies is linked to the possibility of offering health contents to get families more actively involved in clinical pathways. The available literature investigating the benefits derived from hospitals adopting digital technologies reports some positive results for lowering costs, increasing efficiency, improved patient outcomes attributed to electronic medical records which help facilitate clinician decision making, and improving data integrity [19]. The use of m-Health in hospitals can also lead to positive effects on communication between different professionals. For instance, Melby and Hellesø [20] reported that the introduction of an e-message tool at a Norwegian hospital promoted a proactive communication among clinicians and staff. Saddik and Al-Mansour [21] have also reported some positive effects provided by the implementation of a computerized prescriber order entry system in a hospital in Saudi Arabia, in terms of sustained workflow and enhanced nurse–physician communication. However, results are mixed and definitive conclusions on certain aspects as measurements of hospital bed use, equity of access, resource utilization, patient satisfaction and impact on quality of life provider satisfaction and perceived ease of use cannot be clearly established [22]. Regarding the application of digital services in the context of children’s hospital, literature did provide evidence for the positive effects of digital interventions—as serious games and virtual reality—in educating and preparing children for care experiences in different hospital contexts such as surgery and radiology [23,24,25,26]. Although a previous study indicated that families of patients with genetic/neuropsychiatric conditions exhibit a positive attitude toward m-health technologies [27], to the best of our knowledge, there are no studies investigating the application of m-Health systems to improve hospital experience for children with these conditions by supporting their clinical pathways. 

In addition to educational needs regarding health procedures, there is also evidence showing that children and their families need better communication with healthcare personnel in different areas [28]. 

Evidence shows that in Italy family members are often involved in the care of young patients with neuropsychiatric conditions [29,30]. For many families, the assessment process is a critical component of care but may simultaneously be a source of stress [31]. It has been suggested that the involvement of the families in the assessment process can be a central element to improving the overall patient experience [32,33]. The improvement of communication between patients and clinicians provided by the use of m-Health in hospital settings could represent a crucial step towards the increase of active involvement of the families during hospital procedures. 

M-Health can also provide a fundamental contribution to public health systems during this unique historical moment in which we find ourselves. Italy was the first European country to face the coronavirus disease (COVID-19) pandemic in all of its vivid forms. Health systems policies issued significant restrictions on social contacts with the aim of decreasing the exposure to COVID-19 for both patients and providers. Thus, the pandemic has strongly stimulated clinicians to consider alternative service delivery options compatible with social distancing. Within such a framework, new technologies have been considered as a valuable tool to guarantee access to medical services, the World Health Organization (WHO) actually highlighted telemedicine as an essential service in response to the COVID-19 emergency [34]. Digital technologies could indeed provide valuable contributions in large-scale healthcare interventions and relieve pressure on hospitals [35]. Moreover, supporting clinical pathways with m-health can be helpful in reducing in-person contacts and maintaining social distancing. 

We developed an integrated m-Health platform to support clinical pathways and to promote remote contacts between patients and clinicians in the Child and Adolescent Neuropsychiatry Unit of the Bambino Gesù Children’s Hospital, a tertiary care children’s hospital located in Italy. Our unit provides a comprehensive evaluation for diagnostic purposes, including neurodevelopmental, neuropsychological and psychopathological assessment; the assessment process in the day hospital (DH) regimen lasts two or three days according to the estimated clinical needs. Outpatient cooperation and a good ability to focus during the battery of tests is crucial to providing a reliable assessment of patient’s neuropsychiatric and neuropsychological abilities. Thus, it is important to minimize all sources of potential stress in healthcare service delivery, such as long waiting times and the perception of being uninvolved in the ongoing clinical process. These potential stressful factors can contribute to reduced co-operation of patients and their families during the process and could indirectly affect the performance of a child with neuropsychiatric conditions during the assessment. 

In this project, the digital platform Assioma (acronym for the Italian name “ASSIstenza per bambini Ospedalizzati basata su Mobile Application”—Mobile Application-based Assistance for Hospitalized Children) was designed to: improve remote family/doctor communication; enhance family involvement during the DH processes; support automatized data collection, and to support the organization and storage of data. The development of the Assioma platform was performed in compliance with European General Data Protection Regulation (GDPR) [36] and all participants were provided all of the information regarding data management during the informed consent process. This study reports on the usability and user satisfaction of Assioma in a group of families with children attending our unit. First, we illustrate the phase of development of the platform by describing three crucial steps: (i) analysis of the functional requirements; (ii) design of the platform’s architecture; (iii) definition of the functionalities and contents. Then, we report on the results of a pilot test in a small group of participants recruited from within our Unit. 

## 2. Materials and Methods

In total, the development of the Assioma platform took 18 months and included two stages: (1) the development of a digital platform according to an analysis of the functional requirements and needed contents, and the design of the platform’s architecture (2) a pilot study to test Assioma in the DH in the child and adolescent neuropsychiatry unit.

### 2.1. Stage 1. Development

**Figure 1:**
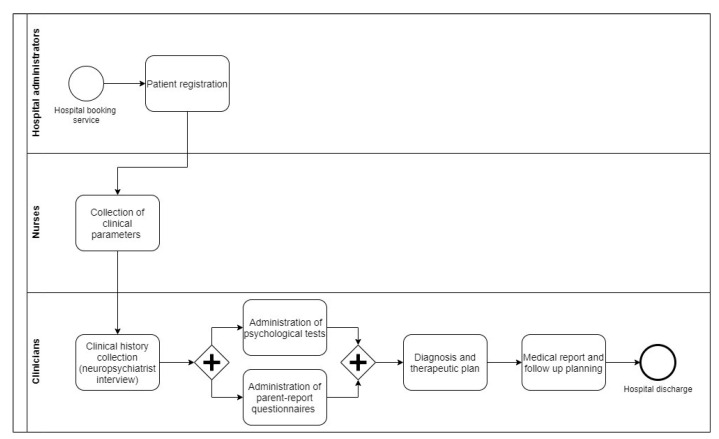
The patient journey of a typical DH access for patients in our Child and Adolescent Neuropsychiatry Unit.

Assioma was developed in accordance with the WHO recommendations on m-Health applications, with particular attention paid to the acknowledged role of m-Health as a complement and an enhancer of health system functions by mechanisms such as accelerating the exchange of information. We also referred to the conceptual foundation provided, regarding accountability, supply, demand, quality, and affordability [37]. The aim of the platform was to meet specific clinical needs for the clinical practice in our mental health service for children and adolescents. The outline of a typical DH clinical pathway is provided in Figure 1. 

As reported in Figure 1, the first interaction between the clinician, the patient and his/her family occurs after checking in to collect their clinical history. This is a crucial passage for the entire evaluation, because caregivers’ interviews are the only way to collect information about the patients that DH staff is not able to observe directly during the assessment. For this reason, it is essential to find the right trade-off between collecting comprehensive and detailed data while completing the task in the shortest amount of time. Moreover, an efficient organization of these data, usually collected in plain language during an interview, is highly desirable for both clinical and research purposes. 

A second aspect to take into account for improving clinical practice is to increase patient and caregiver knowledge and addressing expectations about the DH procedure they will experience. Families can be confused about the organization of clinical procedures before encountering the clinicians who provide instructions (nurses or neuropsychiatrists), and this may sometimes cause delays or ineffective collaboration. Providing simple, clear and child-friendly explanations can be useful to support families at the very beginning of the hospital experience, but could also support them along all their clinical pathways providing further and always-available information in addition to the clinician’s verbal instructions. Moreover, keeping caregivers remotely and constantly updated about the timing of the ongoing procedure may encourage their active involvement during the clinical process. Finally, a third aspect that can improve clinical practice is to enhance caregivers’ awareness of their child’s clinical condition. After a child has received a diagnosis from the clinicians, providing caregivers with supportive and constant, clear information about their child’s condition, symptoms and treatment, can be crucial to guiding them towards an improved awareness, which may be essential to better patient management.

To sum up, the aims of our m-Health platform were to:

(A) Support data collection and organization and to provide effective information;
(B) Promote constant and remote communication between patients, caregivers and clinicians;
(C) Promote families’ active involvement in the DH processing, during and after the DH visits.

#### 2.1.1. Analysis of the Functional Requirements

The analysis of the requirements was carried out in accordance with the American Telemedicine Association guidelines, which recommend a contextual analysis of the clinical, technical and administrative field of the medical framework under investigation [38]. The non-functional requirements (an expression used to refer to the ways in which the system must provide the different functionalities) have been studied. A list of requirements is provided in Table 1.

#### 2.1.2. Design of the Assioma’s Architecture 

**Figure 2:**
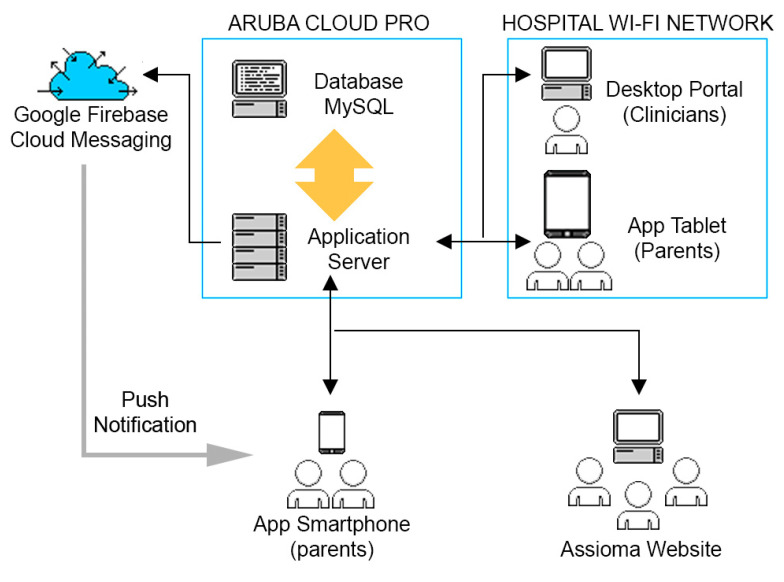
Platform Architecture. The architecture of the Assioma system involves the use of: an infrastructure for Cloud Computing; an infrastructure for sending push notifications—a cross-platform cloud messaging solution; a technology for wireless local area networks (WLAN) using devices based on IEEE 802.11 standards; a public access network for connecting the various devices; a Database Server; an Application Server; different types of clients to access to the different functionalities (Smartphone, Tablet, Personal Computer). The application server is deployed in the cloud and it is a system upon which the applications run handling connections to the database on one side and to the clients (smartphone, tablet, PC) on the other.

The architectural description of Assioma provides an overview of the functional characteristics of the model and the relationships between its different properties (Figure 2).

The policy adopted to adhere to GDPR, implements data security and limits the possibility of external attacks by using: (a) firewalls (b) HTTPS communication protocol; (c) data encryption; (d) token validation and authentication. The Assioma infrastructure had two firewalls: one between the application server and the devices and one between the application server and the MySQL database server. The Assioma system required the use of the HTTPS protocol to ensure data integrity and security in data exchange. Data sent via HTTPS were protected by the transport layer security protocol, which provided three fundamental protection levels: cryptography (used to secure the confidentiality of a message between sender and receiver), data integrity (data cannot be modified or corrupted during transfer) and authentication (protection against man-in-the-middle attacks). An authorization mechanism has also been developed involving the use of a token (based on the Json Web Token standard) for logging into the system.

Interoperability and adherence to the standards used by the Assioma platform guaranteed its extension and maintenance with limited efforts. The Assioma platform was an example of an integrated system through an implementation characterized by: (a) database compliant with the SQL standard; (b) web services based on REST protocol; (c) automatic push information services; (d) multiple devices for content delivery.

#### 2.1.3. Definition of Functionalities and Contents

**Figure 3:**
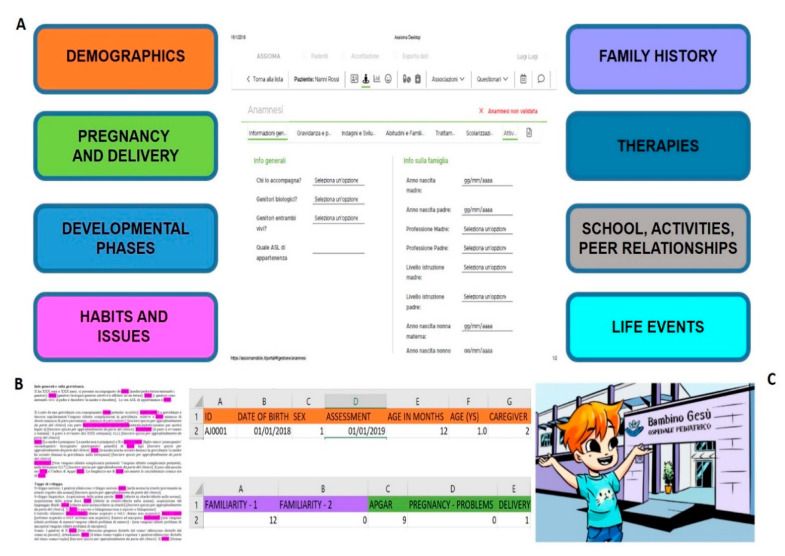
Examples of Assioma contents. (**A**) schematization of the anamnestic questionnaire’s sections and example of a screen. (**B**) examples of data export in text and in Excel of data collected through the anamnestic questionnaire. (**C**) screen of the Video Cartoon 1.

Finally, the application suite was composed of three elements, namely Assioma Desktop, Assioma Parents, and Assioma Diary. Examples of the Assioma contents are provided in Figure 3. Arial font style, size 12, was used for the text included in Assioma Parents and Assioma Diary, based on literature indicating that some font styles have a positive effect on reading abilities for individuals with dyslexia—a population that has been included in our sample [39]:

1) Assioma Desktop was developed for PC, dedicated to clinicians. It contained the information sheet and videos associated with families’ accounts, patient registration and data entry of patients’ physiological parameters functions, possible drugs prescription, medication intake monitoring, DH appointment management and notifications. Through such functionality, clinicians could consult information provided by caregivers filling in the clinical history questionnaire, and then validating it during the interview. Once anamnestic information included in the questionnaire was validated by the clinician, it was automatically exported in text and Excel standard formats. Editable text format was designed accordingly to the format used for hospital discharge, thus only a selected amount of information was included in the editable text form. All the information collected by the anamnestic questionnaire were thus coded and automatically organized in a database format.
2) Assioma Parents was developed for tablet for families during clinical activities. It contained summary information on neurodevelopmental and neuropsychiatric pathologies (epidemiology, etiology, age of onset) to be associated with a participant account at check-in in case of a second visit (according to previous diagnosis) or, in the case of a first visit, at the moment of hospital discharge (according to the diagnosis at the moment of discharge).

Child-friendly video cartoons were created. Cartoon1 explained the DH procedures, while Cartoon2 explained the electroencephalography procedure under deep sedation. 

Caregivers filled out a detailed anamnestic questionnaire with items in 10 different areas: demographics, pregnancy and delivery, developmental phases, previous assessment, habits and issues (e.g., dietary and sleep habits), family history, past and current treatments, school and activities, peer relationships and life events (e.g., potentially traumatic events such as mourning). The questionnaire is available in Supplementary Materials. 

In order to provide logistic information about the ongoing DH procedures and more general material, Assioma Parents also contained a hospital map, and information on the agenda of the ongoing DH visits.

3) Assioma Diary was developed for smartphones, to allow families to use the application outside of the hospital. It contained two of the same functionalities as Assioma Parents (video cartoons and informative sheets), and a push notification service to receive direct communication from clinicians about the ongoing DH procedures (e.g., “appointment at 10.30 in room three after the break”), plus the agenda of future appointments.

### 2.2. Stage 2. Pilot Test

#### 2.2.1. Families and Clinicians’ Enrollment

The enrollment of clinicians was performed in May 2018 during a meeting where the investigators informed the neuropsychiatry staff about the aims and the design of the study, and collected adhesion from psychologists and physicians willing to be involved in the study. 

We enrolled outpatients aged 6–10 years who were referred to our Child and Adolescent Neuropsychiatry Unit for a psychological and psychiatric assessment related to neurodevelopmental and neuropsychiatric conditions. The ownership of a smartphone device with Android operating system represented the third inclusion criterion, given that Assioma was not available for iOS. Exclusion criteria were: less than 6 years of age or older than 10 years; presence of severe neurosensory impairment; clinical suspicion of abuse; absence of an Android-based smartphone device within the household. The study participation was voluntary. 

Patients matching the inclusion criteria for age and diagnostic suspicion were selected among the patients referred to the Unit at the beginning of the planned DH visits. A general outline of the study was provided and informed consent was obtained from all participants. The investigators trained the caregivers in using the mobile app during the first day of the DH programs; caregivers were specifically instructed to supervise the children during the use of Assioma for both tablet and smartphone. 

The project was conducted in accordance with the Declaration of Helsinki and was approved by the hospital Ethical Committee (1804_OPBG_2019). 

#### 2.2.2. Procedure

**Figure 4:**
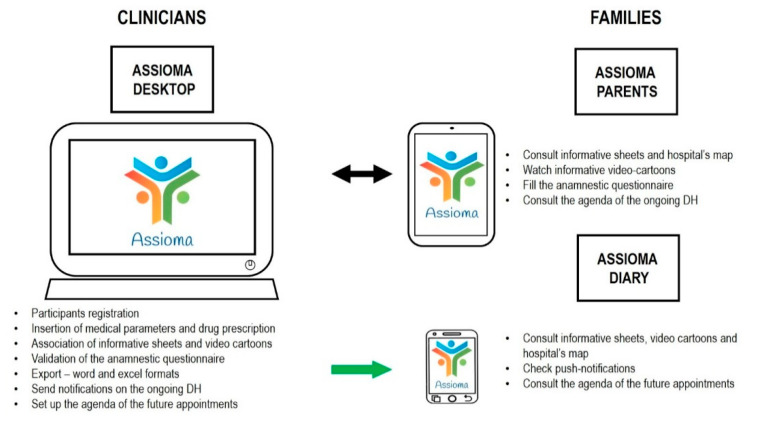
Overview of the users of the three Assioma applications and their tasks. Assioma desktop was only for clinicians, whereas Assioma Parents was for children and their caregivers, with video cartoons designed for children under caregiver supervision. Assioma Diary was an agenda app for caregivers. Bidirectional exchange of information was allowed only between Assioma Desktop and Assioma Parents, when using Assioma Diary caregivers could only access contents associated with their account by the clinicians and check push-notifications.

After patient registration on the Assioma platform by the clinician, a tablet with the Assioma Parents application was provided to the families at the beginning of the DH program, with specific instructions to return it at the end of each day at the DH. Figure 4 summarizes the tasks required of participant and clinicians involved in the study. 

#### 2.2.3. Questionnaires and Data Analysis

The clinical evaluation consisted in a neuropsychiatric interview followed by neuropsychological and psychopathological assessment, including the administration of psychological tests and parent-report questionnaires. 

Once they completed the clinical procedure, families and clinicians involved in the pilot study completed two questionnaires, which included rating their likelihood to use the mobile app and their satisfaction levels from low (disagree) to high (strongly agree). The usability questionnaire for families was composed of 11 items, whereas the version for clinicians was made up of nine items. The satisfaction questionnaire included 8 items for both families and clinicians. At the end of each questionnaire, an additional section for improvement suggestions was added.

## 3. Results

We enrolled 28 children and their caregivers (globally indicated as “families”), and 24 completed the testing. Two families refused to participate because they perceived the tasks required by the testing as too complex and onerous; two families decided to interrupt the testing because of behavioral problems of the children in handling the tablet provided by the investigators. 

A total of 24 families and six clinicians (three psychologists and three physicians) completed the pilot test and filled out the questionnaires. Children were aged between 6–10 years old (mean 7.33, standard deviation 2.3). Eleven children (45.83%) were diagnosed with Specific learning disorder, six (25%) with attention deficit/hyperactivity disorder, four with intellectual disability (16.66%) and three (12.5%) with autism spectrum disorder. 

### 3.1. Usability Questionnaire

Among families, the majority (more than 66%) of caregivers expressed the highest level of approval for the provided explanation of the applications, instructions of use, ease of the log-in, moving among the various functionalities and the completion of the anamnestic questionnaire. The majority of families also expressed scores in the highest range for the items related to the intuitiveness of Assioma for tablets (namely, Assioma Parents) and for the font size and style used. However, mixed results were detected for the items related to the graphical interface (58% of responses gave in the highest score), to the usability of Assioma for smartphone (namely, MyDiary; 62.5% of the responses were in the highest range) and also its navigability (54% of the responses were in the highest range). 

Among clinicians, the majority (more than 66%) of physicians and psychologists expressed the highest level of approval for all the items, with the only exception being the navigability of Assioma Desktop (50% of responses were in the highest range).

Table 2 summarizes the results of the usability questionnaires filled out by caregivers and clinicians. Results are expressed in percentages. 

### 3.2. Satisfaction Questionnaire

Among families, the majority (more than 66%) of caregivers expressed the highest level of approval solely for the perceived improved experience of the DH. Results for the remaining items were mixed. 50% expressed highest scores to evaluate the overall satisfaction of their experience with Assioma. Around half of the families expressed a medium level of satisfaction about the usefulness of the push notifications, video cartoons, and Assioma for smartphone (namely, MyDiary), as well as for the improved communication with clinicians. Finally, 58% gave sufficient scores for the item concerning the usefulness of the information sheets on neuropsychiatric diseases. Of note, the items about the usefulness of MyDiary and information sheets registered quite a high percentage of missing answers (25% and 13%, respectively). 

Among clinicians, the majority (more than 66%) expressed the highest level of satisfaction for the items concerning the usefulness of automatized anamnestic data export in both text and excel formats, as well as for the inclusion of informative contents on neuropsychiatric diseases. Half of the clinicians (50%) expressed the highest level of satisfaction regarding the improved efficiency of DH pathways and improved communication between families and clinicians. Sufficient satisfaction levels for the items concerning the digitalization of the anamnestic questionnaire and overall satisfaction of the Assioma platform were expressed by the majority of clinicians (83% and 67% respectively). 

Table 3 summarizes the results of the satisfaction questionnaires filled by caregivers and clinicians. Results are expressed in percentages. 

## 4. Discussion

To the best of our knowledge, this is the first study to report on the usability and satisfaction of an m-health platform designed to support clinical pathways in a child and adolescent neuropsychiatry unit. Given the literature gap on the application of m-Health platform specifically developed to support clinical pathways in hospital services for children with neurodevelopmental disorders, we designed Assioma to target specific aspects of the DH procedures, namely data collection and organization, family-clinicians communication and families’ involvement in the clinical pathway. 

Overall, the data obtained from this pilot study confirmed an excellent level of usability of the platform, with very high scores obtained from both patients and clinicians, and provided some important suggestions to improve the development of the platform for future application purposes. The excellent usability of a digital platform is an indispensable prerequisite for the effectiveness of m-Health as even applications with high utility may become unlikely to be accepted if they are not user-friendly [40,41]. 

Usability. Most caregivers attributed high scores in evaluating the usability of the different functions of Assioma. The item receiving most heterogeneous answers concerned the intuitiveness of MyDiary (the Assioma app for smartphones). This result could be interpreted by considering the heterogeneity in digital literacy among the involved participants since the use of MyDiary required the ability to download the app from Playstore, installing it and using it during the DH. 

Clinicians attributed very high scores to nearly all of the items, with the only exception being the navigability of Assioma for desktop. This data can be interpreted by recognizing the greater number of functions of the platform the clinicians handled during the pilot study. Moreover, as an integration of the routine clinical procedure, the testing of Assioma during the pilot study was carried out parallel to the use of other software employed in our clinical practice. 

Notably, both groups attributed high scores to the font size used for the platform. This represents an important point considering that our child and adolescent neuropsychiatry unit also includes patients with specific deficits in reading skills; for such patients, the introduction of new devices into clinical practice should consider the application of compensatory measures—such as highly legible characters—to meet their specific needs. 

Differences between the two groups emerged for some functionalities, such as patients’ registration and anamnestic data validation, as well as for the perception of platform navigability and intuitiveness, where clinicians specifically gave scores in low ranges. This divergence can be attributed to possible difficulties experienced by clinicians using the Assioma Desktop. One possible explanation could be the higher number of the platform’s functionalities in the clinician version than those in the patient families’ version. Moreover, the validation of the data from the anamnestic questionnaire consisted in the review of the amount of information collected and then made to flow into the clinical interview that made validation possible. It was therefore a procedure closely connected to clinical practice and, to some extent, additional to routine care. For a future optimization of the application, the clinicians suggested they could benefit from a longer period of training on the use of the platform before starting to use it in the clinical setting. The use of m-Health implicates a reshaping of the conventional clinical pathways [42], and today’s clinicians and healthcare providers are called to drive innovation in clinical practice, especially considering the current pandemic emergency. This process involves the necessity of reflecting on peculiar medicolegal and ethical issues, such as privacy, informed consent, and the doctor-patient relationship [43]. Therefore, an effective training of the healthcare providers should be considered as a crucial step to promote innovation and to bypass resistance to change. 

Regarding satisfaction, in the results obtained from the satisfaction questionnaire, a higher discrepancy emerged for answers provided by caregivers and clinicians for the same items. Overall satisfaction reached the highest levels for 50% of caregivers, but only for 33% of clinicians. As stated previously, former findings had indicated a possible reluctance of clinicians towards the use of m-Health tools within clinical settings and a limited knowledge of information technology [44,45,46,47]. Lapointe and Rivard [48] found that the resistance occurring in the adoption of new m-Health technologies in hospital settings could be fruitfully modulated by the developers. In the present work, we mainly focused on improving, by way of Assioma, the aspects related to the exchange of information between patient families and clinicians. Importantly for the purposes of our study, nearly the 50% of both caregivers and clinicians indicated satisfaction levels in the middle range for the item concerning the improvement of patient-clinician communication. This is in line with literature reporting mixed findings on the improvement of communication between patients and clinicians induced by the introduction of technologies into clinical practice [19]. The heterogeneity of findings could be explained, at least in part, by the variety of the aspects related to patient-clinician communication that can be influenced by the use of m-Health, such as the exchange of information, the adequate response to emotion, the management of uncertainty, the decision-making process, and the enhancement of self-management [49]. However, it must be highlighted that, despite the fact that it represents only one aspect of the multidimensional process of patient-clinician communication, the improvement of information exchange by remote is a crucial goal for clinical pathways in the specific context of a pandemic, where m-Health could be a key ally in promoting social distancing. The absence of answers in the lowest range of satisfaction levels observed in our study could be interpreted as an encouraging suggestion of the noninferiority of the m-Health mediated exchange of information during the DH procedure in child neuropsychiatry services. Finally, we can speculate that the use of the detailed anamnestic questionnaire developed for tablets made it possible for caregivers to focus on some questions which often require more time to respond to, for instance, questions related to life events that may have an impact on the child’s behavior. On the other hand, we could hypothesize that the automatized data collection allowed clinicians to double-check information accuracy, therefore improving the quality of information exchange [50]. Future works should specifically target and investigate the effects of m-Health platforms on the other aspects of communications and should directly compare the efficacy of different remote systems for communication during the DH procedure when face to face communication is discouraged.

Considering group-specific questions, it must be noted that the highest satisfaction ratings for the video cartoons only reached 42% (Video 1) and 29% (Video 2) for families. These findings were in line with our expectations, considering that Video 2 concerned a specific medical procedure, EEG under sedation, thus not all families were interested in receiving information on it. 

The export in text and Excel formats of the anamnestic questionnaire and the information sheets obtained elevated percentages of high scores (more than 80%), in line with our expectations. Data collection, organization and storage—especially anamnestic information—have a central role in the evaluation process in mental healthcare: neuropsychiatric conditions represent very complex phenomena, as individuals included in the same diagnostic category may display a unique combination of factors—both genetic and phenotypic. For this reason, in the past few years great attention has been paid to the possibility of investigating neuropsychiatric condition using big data and machine learning approaches to deal with such high complexity and to help in improving classification of diseases and/or treatment selection, to predict treatment outcomes or test new hypotheses [51,52]. The growing interest in the interaction between technology and mental health led to the possibility of retrieving and analyzing data on multiple health indicators by making patient information available anywhere, anytime [53]. Moreover, it contributed to the creation of a “digital phenotype”, namely the “moment-by-moment quantification of the individual-level human phenotype in situ using data from personal digital devices”, which crucially contributes to the establishment of an ecological and comprehensive approach to mental health measurement [54,55]. 

A further consideration concerns the potential usefulness of the Assioma platform in reducing the in-person contacts between clinicians and patient families. The acquisition of anamnestic information through an m-health system clearly represents an important step towards the optimization of in-person contacts throughout the clinical pathway. Such optimization could allow the clinicians to focus the in-person visit on the direct observation of the patient and physical examination. Therefore, m-health platforms such as Assioma could be valuable tools in situations in which face-to-face visits are not deferrable. This should be then considered an additional way to sustain social distancing in child psychiatry, in partnership with the use of telemedicine. 

To sum up, the optimization of data collection and storage provided by the use of mobile-communication devices could contribute to improving data collection for both clinical and research purposes. The Assioma platform moved in this direction: results from the pilot test indicated that Assioma could effectively support data collection, organization and storage. Moreover, it supported the communication between families and clinicians along the DH pathway, promoting greater involvement by the families in the DH processing procedure and provided a more comfortable experience wothin the DH pathways. Our results confirm and extend previous findings, which have reported a positive attitude toward m-health technologies from families of patients with genetic/neuropsychiatric conditions. We have provided further support for the crucial role of m-Health in child mental healthcare in the relatively unexplored context of the DH clinical pathway

However, some issues must be underlined. The refusal of two families to be involved in the study due to the perceived complexity of the tasks should make us reflect on the possibility of dividing future pilot testing into two parts: one devoted to Assioma Parents and one devoted to MyDiary. Another consideration concerns the interruption of the study related to behavioral issues of two of the children involved at the beginning of the study; in both cases, the children had a diagnostic suspicion (subsequently confirmed at the end of the DH evaluation) of attention deficit/hyperactivity disorder, associated with marked behavioral dyscontrol. This could lead to a change in the exclusion criteria for further studies. A further limitation of the study was the lack of development for iOS, which restricted the number of families recruitable for the pilot testing. 

Clinicians suggested investigating the usability of Assioma for the outpatient procedures of our Unit, where the functionality of the Assioma anamnestic questionnaires would be highly useful. A further step towards the digitalization of clinical pathways could be the administration of the digital version of some of the open-source questionnaires used in the assessment process, and their automated scoring, thus allowing the clinician to further optimize the time of the evaluation process. Moreover, it would be desirable to design additional video cartoons to introduce other medical procedures, such as blood sampling, which is a common procedure for some patients. Finally, considering the gap in satisfaction levels between caregivers and clinicians, the involvement of families (for instance, through focus groups) could be helpful in designing additional platform functionalities, for the families’ side. Conversely, for the clinicians’ side, planning for a longer training time could help them to reach an optimum level of familiarity for the best integration between platform use and clinical activities.

Despite the encouraging results, we must underline that this study was conducted in a single center on a small sample size. The nature of the study was chosen to provide detailed qualitative information useful for planning future larger studies. Not only would large multicenter studies be helpful to better and more precisely assess the impact of mHealth tools in the neuropsychiatry setting, but the heterogeneity of the population under study would also help in evaluating their applicability based on cultural differences and age groups. These studies will be necessary to definitely confirm our results.

The use of digital tools is also relevant to regulation for data management. All digital applications developed in the EU should comply with GDPR [36]. Our study was conducted in compliance with current regulations and all information about data management was included in the informed consent for participating. It is worth noticing that beyond GDPR compliance, the evaluation of digital tools for clinical uses is currently conducted with different approaches at the international level. Regulations for authorization of digital tools for the clinic, for example, vary from the US to the EU. Moreover, in Europe, some countries like Germany, recently issued specific regulations for digital tools for clinical use. Future studies should be conducted taking into account the evolution of regulations for digital health.

## 5. Conclusions

The data obtained from this pilot study constitute a promising background for further improvement and validation studies of the Assioma platform as a medical device potentially usable in different contexts of clinical pathways, such as the DH and outpatient regime, with the aim of improving patient-doctor communication, to support clinical pathways in the hospital and to potentially sustain social distancing in this a unique historical moment of the COVID-19 pandemic. The Assioma platform therefore could pave the way towards new digitalization procedures for clinical procedures, with important expected benefits for optimizing clinical times, increasing compliance, improving clinical efficiency and supporting safety procedures through social distancing during pandemic emergencies.

## Figures and Tables

**Table 1:** Functional and nonfunctional requirements of the platform.

**Functional Requirements**	**Non-Functional Requirements**
Guide to the facility Acceptance and registration 1. A feature that enables univocal association of a code to every patient into the system; 2. Authentication of user whenever he/she logs into the system; 3. Enforce automatic logoff. Role and times 1. Two types of users’ accounts: clinicians and families; 2. A feature that enables clinicians to create the agenda of the planned activities of the DH; 3. A feature that enables families to visualize the agenda of fu-ture appointments and the schedules of the DH activities. Procedures 1. A function that enables families to have information to navi-gate through the building and on hospital facilities; 2. A page with informative contents (video cartoons) on the steps for specific instrumental examinations (electroencephalography).	Performance and scalability The software should be an adaptable tool that can be improved in its functions and capacity according to users’ requests and needs.
	Interoperability The software should be able to communicate with other software.
	Portability The software should be able to be used in different hospital contexts and by different users.
Communication and assistance Medical History 1. Different users’ dashboard pages (clinicians, families); 2. A page with a list of anamnestic questions focused on neuro-psychiatric and psychological development; 3. A feature that enables users to explore different sections of the anamnestic questionnaire; 4. A feature that enables users (families) to fill the anamnestic questionnaire; 5. A feature that enables users (clinicians) to visualize and validate anamnestic data provided by families. Patients communications and notifications 1. A push notification service that enables prompt information exchange from clinicians to families about the DH steps and procedures; 2. Pages with educational contents about: (i) neuropsychiatric conditions and treatment; (ii) the DH organization and clinicians’ roles (e.g., psychologists, nurses); 3. A feature that enables clini-cians to associate educational contents to specific users’ account; 4. A feature that enables families to visualize and explore the associated educational contents (patient education).	Privacy and security The software should guarantee adequate privacy and security protections for sensitive data exchanged remotely (compliance with GDPR).
	Accessibility The software should include different devices for a wide range of users, allowing sufficient response time also for the slowest users.
Diagnostic Survey management 1. A feature that enables remote anamnestic data acquisition by clinicians; 2. A function that enables the automatized conversion of data collected through the anamnestic questionnaire into a text for-mat. Reporting 1. A function that enables accessibility and extraction for statistical analysis through the automatized conversion of data collected into a database. 2. A function that enables clinicians to complete, edit, add, and delete different fields in the resume file for the DH discharge.	Usability The software should be user-friendly, allowing an interaction perceived as effective by the users. It should also re-quire minimal training time for using the system.
	Supportability
	The software should be: (i) easy to install and configure: (ii) cost-effective to maintain.
	Manageability The software should support system admin in troubleshooting problems.

**Table 2:** Percentages of usability for each statement.

**Usability-Caregivers Questionnaire**	**Strongly Agree**	**Moderately Agree**	**Disagree**	**Missing**
The explanations provided for the use of Assioma were clear.	87.5% (21)	12.5% (3)	-	-
I would have preferred more detailed explanation for using Assioma.	4% (1)	21% (5)	75% (18)	-
It was easy to perform log-in.	79% (19)	17% (4)	-	4% (1)
The graphical interface of the platform was pleasant.	58% (14)	42% (10)	-	-
The contents of the platform were clear and easy to understand.	79% (19)	21% (5)	-	-
It was easy to move from one section to another of Assioma for tablet.	67% (16)	33% (8)	-	-
It was easy to complete the anamnestic questionnaire.	71% (17)	29% (7)	-	-
It was easy to move from one section to another within Assioma for smartphone.	62.5% (15)	29% (7)	-	8.5% (2)
Assioma for tablet was intuitive and easy to navigate.	75% (18)	25% (6)	-	-
Assioma for smartphone was intuitive and easy to navigate.	54% (13)	29.5% (7)	4% (1)	12.5% (3)
I am satisfied of the font size and style used in Assioma application.	83.5% (20)	12.5% (3)	-	4% (1)
**Usability-Clinicians Questionnaire**	**Strongly Agree**	**Moderately Agree**	**Disagree**	**Missing**
The explanations provided for the use of Assioma were clear.	100% (6)	-	-	-
I would have preferred more detailed explanation for using Assioma.	83% (5)	17% (1)	-	-
Patients’ registration was easy.	67% (4)	33% (2)	-	-
The graphical interface of the platform was pleasant.	83% (5)	17% (1)	-	-
I was satisfied with the font size of Assioma.	100% (6)	-	-	-
The contents were clear and easy to understand.	100% (6)	-	-	-
It was easy to move from one session to another of Assioma for PC.	50% (3)	50% (3)	-	-
The validation and integration of the medical history questionnaire was easy.	67% (4)	33% (2)	-	-
Assioma for PC was intuitive and easy to navigate.	67% (4)	33% (2)	-	-

**Table 3:** Percentages of satisfaction for each statement.

**Satisfaction-Caregivers Questionnaire**	**Strongly Agree**	**Moderately Agree**	**Disagree**	**Missing**
Assioma made the Day Hospital experience more comfortable.	67% (16)	33% (8)	-	-
The Day Hospital notifications were helpful for hospital experience.	34% (8)	54% (13)	4% (1)	8% (2)
The vision of the video cartoon on the Day Hospital procedure was useful.	42% (10)	50% (12)	4% (1)	4% (1)
The vision of the video cartoon on the EEG procedure was useful.	29% (7)	50% (12)	12% (3)	9% (2)
The vision of the information sheets was useful.	25% (6)	58% (14)	4% (1)	13% (3)
Assioma for smartphone was useful.	21% (5)	46% (11)	8% (2)	25% (6)
Assioma improved the communication with clinicians.	42% (10)	54% (13)	-	4% (1)
I am globally satisfied of Assioma platform.	50% (12)	46% (11)	-	4% (1)
**Satisfaction-Clinicians Questionnaire**	**Strongly Agree**	**Moderately Agree**	**Disagree**	**Missing**
The digitalization of the anamnestic questionnaire was useful for the clinical practice.	17% (1)	83% (5)	-	-
The export of the anamnestic questionnaire in text format was useful for the clinical practice.	83% (5)	17% (1)	-	-
The export of the anamnestic questionnaire in excel format was useful for the clinical practice.	100% (6)	-	-	-
The inclusion of informative contents on neuropsychiatric diseases was useful for the clinical practice.	83% (5)	17% (1)	-	-
The use of Assioma made the Day Hospital procedure more efficient.	50% (3)	50% (3)	-	-
Notifications on the Day Hospital activities were useful for the clinical practice.	33% (2)	33% (2)	17% (1)	17% (1)
Assioma improved the communication between patients/parents and clinicians.	50% (3)	50% (3)	-	-
I am globally satisfied of Assioma platform.	33% (2)	67% (4)	-	-

## Data Availability

The data presented in this study are available on request from the corresponding author. The data are not publicly available due to the consent provided by participants on the use of confidential data.

## Supplementary Materials

The following are available online at https://www.mdpi.com/1660-4601/18/5/2758/s1, Anamnestic Questionnaire S1.
