# Supplementary material for: Mobile-Health Technologies for a Child Neuropsychiatry Service: Development and Usability of the Assioma Digital Platform
Source: Int J Environ Res Public Health. 2021 Mar 9;18(5):2758. doi: 10.3390/ijerph18052758 (PMC7967477; doi:10.3390/ijerph18052758)
Supplement: Supplementary file 1 [file ijerph-18-02758-s001.pdf]

Supplementary Material

Anamnestic Questionnaire

1. Date of Birth.

General Information

2. Who accompanies X? ____________
3. Biological or adopted parents? YES-NO
4. Are both parents alive? YES-NO
    - Please, specify ______________
5. How tall is X?
6. How much does X weigh?
7. Local healthcare ____________

Family and Demographic Information

8. Date of birth - mother:
9. Date of birth - father:
10. Occupation - mother:
    - Unemployed
    - Scientific/educational/medical/highly specialized professional (physician, researcher, teacher, psychologist, engineer etc…)
    - Office worker
    - Entrepreneur/businessman
    - Health professional (nurse, speech therapist, sociomedical operator, obstetrician etc…)
    - Service industry/salesperson
    - Farmer
    - Artisan
    - Laborer
    - Member of armed forces
    - Manager/director/executive (public administration)

- Manager/director/executive (private)
- Artist/athlete

11. Occupation - father:
    - Unemployed
    - Scientific/educational/medical/highly specialized professional (physician, researcher, teacher, psychologist, engineer etc…)
    - Office worker
    - Entrepreneur/businessman
    - Health professional (nurse, speech therapist, sociomedical operator, obstetrician etc…)
    - Service industry/salesperson
    - Farmer
    - Artisan
    - Laborer
    - Member of armed forces
    - Manager/director/executive (public administration)
    - Manager/director/executive
    - Artist/athlete

12. Educational level - mother: middle license / high school diploma / degree/ PhD or other high specialization
13. Educational level - father: middle license / high school diploma / degree/ PhD or other high specialization
14. Date of birth – maternal grandmother: ____________
15. Date of birth – maternal grandfather: ____________
16. Date of birth – paternal grandmother: ____________
17. Date of birth – paternal grandfather: ____________
18. Are parents divorced? YES-NO
    - If yes, the child lives with: mother-father
    - If yes, are parents remarried? NO – BOTH – MOTHER – FATHER

Pregnancy and Delivery

19. Was it the first pregnancy? YES-NO
20. X is:
    - Only child;
    - Firstborn;
    - Second-born;
    - Third-born;
    - Fourth-born;
    - Twin;
    - Other (please, specify _____________)
21. How many children do you have? ____________
    - Other 1: M-F; Firstborn; Second-born; other
    - Other 2: M-F; Firstborn; Second-born to; other
    - Other : M-F; Firstborn; Second-born; other
22. Conception was: natural/assisted.

    If assisted, specify:
    - Transvaginal ovum retrieval
    - Embryo tranfer
    - Intracytoplasmic sperm injection - Icsi
    - Testicular sperm retrieval
    - Cryopreservation
    - Gamete intrafallopian transfer - Gift
    - Zygote intrafallopian transfer - Zift
    - Tubal embryo transfer - Tet
    - TEsticular Sperm Aspiration - Tesa
    - Microsurgical Epidydimal Sperm Aspiration – Mesa

23. Were there complications in pregnancy? YES-NO

    If yes, specify:

- Risk of abortion
- Risk of Pre-term labor
- Risk of induced delivery

24. Which week of pregnancy did the birth occur? N° ____
25. Childbirth…
    - Natural
    - Cesarean section
26. If natural…
    - Was forceps used? YES-NO
    - Was vacuum used? YES-NO
    - Was it necessary to induce the birth with a drug (oxytocin)? YES-NO
27. If caesarean section, why? Please specify: _________
28. Was X ever hospitalized immediately after birth? YES-NO
    - If yes, please specify: ____________
29. Birthweight: _________gr
30. Length at birth: _______cm
31. Head circumference at birth: _____ cm
32. Apgar: N° __-__

Previous Medical Examinations

33. Has X ever had an EEG?
    - If yes, in which year? ____________
34. Has X ever had an MRI?
    - If yes, in which year? ____________
35. Has s X ever had an audiometer examination?
    - If yes, in which year? ____________
36. Has X ever had a neuropsychological, psychological or neuropsychiatric assessment?
    - If yes, in which year? ____________
37. Has X ever had genetic analyses?
    - If yes, in which year? ____________

Developmental Stages

38. Feeding: breastfeeding – artificial milk - mixed
39. Was Gastroesophageal Reflux present during breastfeeding? YES-NO

40. How many months did the weaning take place?
41. How many months did X walk? ____________
42. Did/does X use coded signals to communicate (for instance, “bye bye”, “yummy” ecc…)? YES-NO
43. Did/does X use gestures to indicate objects, persons or events in the environment? YES-NO
44. Did/does X use gestures while playing, as getting the hand close to the ear for “telephone”? YES-NO
45. How many months did X say the first words?____________
46. How many months did X say the first sentences? ____________
47. Did language develop regularly? YES-NO
48. Is the child exposed to languages other than Italian? For example: he/she speaks Italian at school, but at home he/she mainly speaks another language. YES-NO
49. Is the child potty-trained (day)? YES-NO
    - If yes, how old were he/she? ____________
50. Is the child potty-trained (night)? YES-NO
    - If yes, how old were he/she? ____________
51. Does he/she have enuresis? YES-NO
52. Does he/she have encopresis? YES-NO
53. Has sleep always been regular? YES-NO

Habits and Issues

54. Is sleep regular now? YES-NO
55. Does X sleep in his/her own bed and in his/her bedroom? YES-NO
56. What time does X go to sleep?
    - between 9pm and 10pm
    - between 10pm and 11 pm
    - between 10pm and 12 pm
    - after midnight
57. How many hours do you sleep?
    - Less than 5 hours/night
    - 5-6 hours/night
    - 7-8 hours/night
    - More than 8 hours

58. Is his/her diet regular? YES-NO

If no:

- He/she eats too much
- He/she eats too little
- he only eats some foods

59. X's diet is:

- vegetarian
- veggie
- mixed

60. Does X routinely take one or more of the following products?

- food supplements
- diuretics
- laxatives
- digestive
- herbal products
- energy drinks and energizers

61. Does X have gastrointestinal problems? YES-NO.

If yes, please specify:

- constipation
- vomit
- acid reflux
- diarrhea

62. Does X tire easily? YES-NO
63. X has or has had recurrent ear infections (> 6 per year)? YES-NO
64. Does X frequently have phlegm? YES-NO
65. Only for females: did she have her first menstruation? YES-NO

- If yes, please specify the age at first menstruation: ____________

Family History

66. Do you have cases of neurodevelopmental conditions in your family? For instance intellectual disability, autism spectrum disorder, speech problems etc… Please, consider only close family, as X parents, brothers, grandparents and NOT in-laws. YES-NO

If yes, what cases of neurodevelopment problems are or have been present in the maternal line of kinship?

- Autism
- Intellectual disability
- Communication Disorders
- Specific Learning Disorder
- Attention deficit/hyperactivity disorder
- Genetic syndromes

If yes, what cases of neurodevelopment problems are or have been present in the paternal line of kinship?

- Autism
- Intellectual disability
- Communication Disorders
- Specific Learning Disorder
- Attention deficit/hyperactivity disorder
- Genetic syndromes

67. Do you have cases of psychiatric conditions in your family? For instance anxiety, depression etc… Please, consider only close family, as X parents, brothers, grandparents and NOT in-laws. YES-NO

If yes, what cases of psychiatric conditions are or have been present in the maternal line of kinship?

- Depression/bipolar/ mood disorders
- Feeding and eating disorders
- Anxiety Disorders
- Disruptive, Impulse-Control, and Conduct Disorders
- Psychosis/schizophrenia
- Tic
- Substance-Related and Addictive Disorders

If yes, what cases of neurodevelopment problems are or have been present in the paternal line of kinship?

- Depression/bipolar/ mood disorders
- Feeding and eating disorders
- Anxiety Disorders
- Disruptive, Impulse-Control, and Conduct Disorders
- Psychosis/schizophrenia
- Tic
- Substance-Related and Addictive Disorders

Past and Current Treatments

68. Did X ever receive any kind of diagnosis in the past? YES-NO
    - If yes, specify: ____________
69. Does X take medications? YES-NO
    - If yes, specify: ____________
70. Did X take any medications in the past? YES-NO.
    - If yes, specify: ____________
71. Ongoing treatments:
    - Psychological/behavioral: YES-NO. If yes, how many hours/week? ____________
    - Speech therapy: YES-NO. If yes, how many hours/week? ____________
    - Psychomotricity: YES-NO. If yes, how many hours/week? ____________
    - Other: YES-NO. If yes, how many hours/week? ____________
72. Past treatments:
    - Psychological/behavioral: YES-NO. If yes, how many hours/week? ____________
    - Speech therapy: YES-NO. If yes, how many hours/week? ____________
    - Psychomotricity: YES-NO. If yes, how many hours/week? ____________
    - Other: YES-NO. If yes, how many hours/week? ____________

School

73. Did X attended nursery? YES-NO
74. X is currently attending:
    - Preschool

- Primary school
- Secondary school
- High-school

75. Is X willing to go to school? YES-NO
76. Does X part peacefully when you leave him/her at school? YES-NO
77. Do teachers refer any learning difficulty? YES-NO
78. Do teachers refer any behavioral problem? YES-NO
79. Do teachers refer attention problems? YES-NO
80. How is academic performance?
    - good
    - sufficient
    - poor
81. Have there been school failures (rejection or recoveries)? YES-NO

    If yes,

- Failure: academic year: __________
- Recovery: school subject_________ academic year: __________

82. What is the most difficult school subject for X?
    - Matematica
    - Literature
    - English/French
    - Other
    - All
    - No one
83. Does X have a suppor teacher? YES-NO.
    - If yes, please specify the number of hours/week: __________

Activities and Socialization

84. Is X involved in sports or physical activities? YES-NO

    If yes, specify: __________ how frequently?
    - Every day
    - 2-3 times/week
    - 4-5 times/week

85. Is X involved in other activities? YES-NO

    If yes, specify: __________

86. Does X watch TV? YES-NO

    If yes, how many hours/day?

    - About half an hour
    - About 1 hour
    - About 2 hours
    - More than 2 hours

87. Does X use smartphone and/or tablet? YES-NO

    If yes, how many hours/day?

    - About half an hour
    - About 1 hour
    - About 2 hours
    - More than 2 hours

88. Does X have good relationships with peers? YES-NO
89. Does X have good relationships with adults? YES-NO
90. Does X have good relationships within family? YES-NO

Life Events

91. Have there been any particularly critical events in X’s life? YES-NO.

    If yes:

    - Mourning(s)
    - Parents’ divorce
    - Fight or separation from friends
    - Car accident
    - Household accident
    - Illness of relatives or friends
    - Natural disaster (earthquake etc…)

92. Have there been other events that you think have significantly affected X's life? YES-NO. If yes, specify: ____________________

93. Why does X come to visit? ____________________

94. Who suggested this visit for your child?

- School
- Family doctor
- Psychologist
- Speech therapist
- Other kind of physician
- Informal suggestion
- We came here spontaneously
